# Video and Telephone Telehealth Use and Web-Based Patient Portal Activation Among Rural-Dwelling Patients: Retrospective Medical Record Review and Policy Implications

**DOI:** 10.2196/67226

**Published:** 2025-08-29

**Authors:** Meghan Rowe Ferrara, Gina Intinarelli-Shuler, Susan A Chapman

**Affiliations:** 1 Department of Social and Behavioral Sciences, School of Nursing, University of California, San Francisco, 490 Illinois Street, Floor 12, Box 0612, San Francisco, CA, 94143, United States, 1 415 476 3047

**Keywords:** health care access, patient demographics, patient portal, rural, rural health, telehealth, video visit, telephone, demographic analysis, policy, health disparities, audio, medical record, United States, adult, rural-dwelling patients

## Abstract

**Background:**

Telehealth may redress rural health care shortages in the United States and improve related rural health disparities. However, following the expansion of telehealth related to the COVID-19 pandemic, telehealth use has been lower among rural populations compared to urban populations. Certain populations are also more likely to use audio-only telehealth, with implications for care quality.

**Objective:**

The aim of this study is to describe demographic and telehealth use characteristics of a population of rural-dwelling adult patients and explore relationships of these characteristics with patients’ level of rurality and with modality of patients’ most recent telehealth encounter.

**Methods:**

We conducted a retrospective medical record review of adults who lived in rural California zip codes and used telehealth at an urban medical center from December 2021 to December 2022. Rural-Urban Commuting Area codes were used to assign rurality and group patients by 3 levels of rurality. Telehealth visits were defined as video-enabled and telephone encounters. Variables included age, race or ethnicity, preferred language, payer, web-based patient portal activation status (as proxy for digital health literacy), video or telephone modality, and visit provider. Chi-square and Fisher exact tested associations of demographic and encounter characteristics with patient level of rurality and telehealth encounter modality.

**Results:**

A total of 9359 patients were included. Telehealth patients living in the most rural zip codes were older, and a higher proportion were White compared to those in less rural zip codes. Patients who were American Indian, Asian, Black, and Latino together comprised 18.8% (n=1760) of the sample, lower than that in rural California counties. Video visit use was significantly lower among patients who were 65 years of age or older (n=3183, 91.3% vs n=5507, 93.8% for younger than 65 years; *χ*^2^_1_=19.3; *P*<.001), Latino race or ethnicity (n=1229, 90.9% vs n=6078, 93.4% among White patients; *χ*^2^_3_=12.0; *P*=.008), primary Spanish speakers (n=336, 87.7% vs n=8305, 93% among English speakers; Fisher exact, *P*<.001), and publicly insured (Medicare: n=3843, 91.7%; Medicaid: n=1717, 92.2%; privately insured: n=3130, 94.7%; *χ*^2^_2_=27.9; *P*<.001). Patient portal activation was lower among Latinx patients (n=1183, 87.5% vs n=6099, 93.7% among White patients), Spanish speakers (n=295, 77% vs n=8241, 92.3% among English speakers; Fisher exact: *P*<.001), and Medicaid patients (n=1635, 87.8%; Medicare: n=3802, 90.7%; privately insured: n=3140, 95%; *χ*^2^_4_=106.3; *P*<.001).

**Conclusions:**

Findings substantiate concerns of rural telehealth access disparities, particularly among patients who are older, of minoritized race or ethnicity, and Spanish-speaking. Ongoing research is needed to understand how underserved rural populations use telehealth. To address telehealth use disparities, policy should address patient-level barriers by supporting measures such as health care navigation resources, culturally tailored telehealth patient outreach, digital access assessment, and patient digital education. Evidence-based telehealth reimbursement policy is essential to support access and address provider-side barriers.

## Introduction

In the United States, rural populations experience worse outcomes related to the most common health conditions as well as a higher burden of morbidity and mortality compared to urban populations [1-5]. These rural health disparities are often more pronounced among rural populations of color, who make up about 20% of rural US residents [3,6,7]. Rural health disparities negatively impact rural social systems and prevent rural communities from thriving [8].

A major contributor to rural health disparities is limited health care access, a chronic issue with multifaceted causes, including structural factors that constrain the overall availability of health care in rural areas [4,8-10]. Long-term trends in health care organization, health system affiliation, and rural economies have resulted in the reduction of services or closure for hundreds of rural health care facilities nationwide, including hospitals, nursing homes, and pharmacies [3,8,11]. Maldistribution of the health care workforce between urban and rural areas also limits rural health care access with severe shortages of rural health care providers, including in primary care but most extreme among specialist providers [4,12-14].

The digital delivery of health care using communication technologies, broadly known as telehealth, may improve rural health care access by connecting rural patients to remote health care providers where they already practice [15-17]. Despite this promise, widespread scale-up of telehealth provision was not realized until the COVID-19 pandemic, which necessitated an abrupt shift away from in-person care in March 2020. Subsequent telehealth reimbursement expansions by both the Centers for Medicare and Medicaid Services (CMS) and private insurers resulted in rapid, dramatic increases in the share of health care encounters conducted via telehealth [18-20].

Since the declaration of the COVID-19 Public Health Emergency (PHE), however, a picture has emerged of the mixed impact this rapid expansion has had on telehealth access. While the increase in telehealth appears to have improved access for some patients [18], evidence has shown that telehealth use during the COVID-19 PHE followed historical health care and telehealth access disparity trends [21], revealing lower use among patients who are rural dwelling [18,20,22,23], lower income [20,22], uninsured [24], and belong to certain race or ethnicity groups [18,22]. Furthermore, the use of video versus audio-only telehealth modalities introduces a new dimension to access concerns. It remains unclear whether audio-only or telephone visits support the same care quality as video visits [25-28], and video use has been shown to vary by age [28-32], income [31,32], education [30-32], insurance [28,29,31,32], race and ethnicity [28-33], patient language [28,30,31,33], rurality [28,33,34], and area broadband availability [28,31]. However, studies report sometimes contradictory telehealth and video visit use across patient characteristics, and findings vary by region [18,22,32], health care setting, specialty, and diagnosis [29,30,34].

Given the complexity of telehealth use, further research is needed to more fully understand how specific patient populations are using telehealth. This is of particular importance in specialized health care settings, where access barriers may be more pronounced, and among populations already at risk of access disparities, such as rural populations and populations of color. The purpose of this paper is to describe the demographic characteristics of a population of rural-dwelling adults in California who used telehealth services at a large urban medical center and to describe visit characteristics of these patients’ most recent telehealth encounters, including video or telephone modality. We also explore the relationship of patient demographic and telehealth encounter characteristics with the degree of patient rurality and with the modality of patients’ most recent telehealth encounter. Our analysis expands on existing analyses of rural telehealth use by applying 3 levels of patient rurality to assess patients’ demographic and telehealth use characteristics. We conclude with a discussion of the policy implications of our findings.

## Methods

### Data and Study Setting

Data in this retrospective study were obtained from the electronic health records (EHRs) of a large health system providing diverse specialty care, located in a major urban center in California. Data from patients with telehealth encounters at the health system in the 1-year period from December 2021 to December 2022 were included in this study. We selected this time frame as a later phase of the COVID-19 PHE when telehealth care was well-established, but in-person restrictions had been loosened, and telehealth use had settled from its peak pandemic levels. For this study, telehealth visits were defined as video-enabled and telephone encounters between a patient and any provider type.

### Ethical Considerations

This study was approved by the University of California, San Francisco Institutional Review Board (review #22-35996). As a retrospective medical record review of deidentified patient data that had been previously collected as part of clinical care and quality improvement, it was deemed exempt from an informed consent process and HIPAA (Health Insurance Portability and Accountability Act) authorization. Data sharing was not included in institutional review board approval.

### Study Population

All adult patients (≥18 years) in the health system residing in a rural California zip code (see Assigning Rurality section) who had at least 1 telehealth encounter in the study period (December 2021 to December 2022) were included in the dataset (N=9359). The study population was drawn from a geographically diverse area of California and included residents of zip codes in a radius of hundreds of miles from the health center. According to our identified zip codes, rural patients comprised 4% of total telehealth patients at the health center during the study period.

### Assigning Rurality

Rural patients were identified using Rural-Urban Commuting Area (RUCA) codes [35] zip code approximations from the Washington, Wyoming, Alaska, Montana, Idaho Rural Health Research Center [36]. Along with their wide use in health services research, we chose RUCA codes because they provide a more granular breakdown of rurality than alternative systems that are based at the county level, such as Rural-Urban Continuum Codes. Given high levels of within-county variation in population density and distribution in many California counties, county-level classifications may collapse important rural-urban distinctions. RUCA codes avoid this by classifying based on the smaller scale of US Census tracts [37]. RUCA codes are assigned to US Census tracts based on population density, measures of urbanization, and daily commuting flows. The Washington, Wyoming, Alaska, Montana, Idaho Rural Health Research Center zip code database combines RUCA values from census tracts that comprise specific zip code areas [37], allowing researchers to apply RUCA codes to more easily accessed patient zip codes.

**Figure 1.**
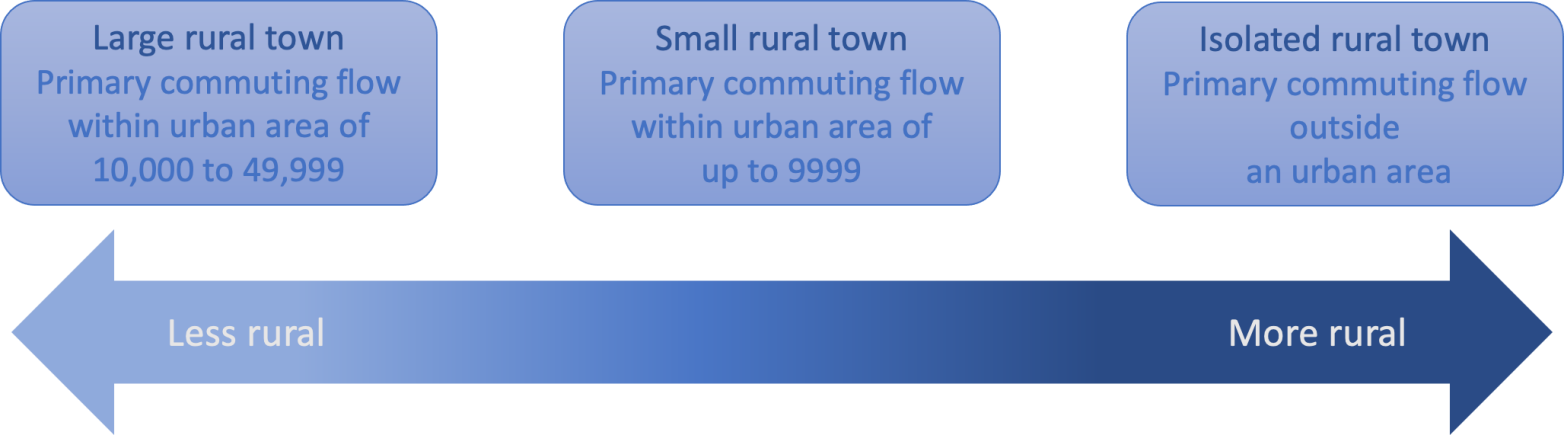
The 3 levels of rurality used to group included patients, with commuting flow population density and relative rurality of each group. Developed based on the “categorization A” [38] organization of Rural-Urban Commuting Area codes [37] suggested by the Washington, Wyoming, Alaska, Montana, Idaho Rural Health Research Center.

We used a 4-level urban-rural categorization of RUCA codes [38]: urban; large rural city or town (micropolitan), the most populous or least rural level; small rural town; and isolated small rural town, the most rural level. All California zip codes in the 3 rural categories were included (Figure 1). These RUCA groupings allowed us to analyze demographic and telehealth encounter characteristics of a diverse rural population with more nuance, reflective of meaningful measures of rural population density and resource proximity.

### Variables

#### Patient Demographic Variables

We extracted the following patient demographic characteristics (Table 1): zip code, age, sex, race or ethnicity, preferred language, payer, and patient portal activation status. Each patient was then grouped by zip code into 1 of the 3 rurality levels described earlier. EHR data at the health system do not have separate variables for race and ethnicity (eg, Hispanic ethnicity); we used labels in the EHR (eg, Latinx). Some categories of race or ethnicity and preferred language had very few observations in the small rural town and isolated rural town levels. To enable association tests, we combined categories within these variables, as described in Table 1.

Patients of the health system can make use of a web-based patient portal account to securely access personal health information and services such as provider messaging. Patient-portal activation status was collected as a proxy for digital health literacy or comfortability. At the study health system, a patient portal account is not required for video visits. Patient portal activation status is not a complete measure of digital health literacy or comfortability and cannot fully capture factors such as broadband access, device ownership, or patient preferences, such as trust in digital platforms. Nevertheless, this measure may reflect a patient’s level of engagement or comfort with digital health tools, and as a proxy measure, it has been shown to provide valuable insight in the literature [31,34,39,40]. Finally, payer or insurance type was categorized as either Medi-Cal (California’s Medicaid plan, a joint federal and state program providing health coverage to low-income individuals), Medicare (a federal program providing health insurance to those aged 65 years or older and those younger than 65 years with certain disabilities or health conditions, regardless of income level), or other insurance (including commercial health plans, the predominant form of health insurance in the United States, often provided through an individual’s employer).

**Table 1.** Patient demographic and telehealth encounter variables in the dataset.

Variable name	Description
Patient demographic variables	
Zip code	US zip code of patient’s residence address.
Level of rurality	Patients’ zip codes were used to group patients into 1 of 3 rurality levels (from least to most rural): large rural town, small rural town, or isolated rural town.
Age	Exact age at time of first telehealth encounter and dichotomous age, younger than 65 years and 65 years or older.
Sex	Four categories: female, male, unknown, or nonbinary. Unknown and nonbinary had too few observations to support tests of association and were excluded from analyses.
Patient-identified race or ethnicity	Four categories included in analyses: White, Latinx, unknown or declined, and combined other. Categories included in the combined other were Asian, Black or African American, Native American or Alaska Native, Native Hawaiian or other Pacific Islander, Southwest Asian and North African, multirace or ethnicity, and other.
Preferred language	Three categories included in analyses: English, Spanish, and other. Other included 21 additional languages.
Payer	Type of health insurance, 3 categories: Medicare, Medi-Cal (California’s state Medicaid program), and other insurance. Other included commercial health plans, Covered California insurance^a^, self-pay^b^, and several other less common insurance options.
Patient portal activation status	Activated: portal account set up completed and does not indicate recency of account creation or access, pending activation: patient issued an activation code but had not yet completed account set-up, and inactivated: account creation not completed before the activation code expired after 30 days.
Telehealth encounter variables	
Telehealth modality	Mode of telehealth delivery (video or telephone) for each patient’s single most recent telehealth encounter in the study period.
Provider	Health care professional charted for the telehealth encounter: physician, nurse practitioner, physician assistant, and other. Other included resource providers, including counselors and chaplains, resident physicians, and all other provider types.
Specialty area	Primary care: included primary care and family medicine; medical specialties: any nonsurgical specialties; surgical specialties: including surgical oncology; oncology and cancer center care: all nonsurgical cancer-related care; and women’s and maternal health: including fetal health and neonatology.

a California’s subsidized health insurance marketplace created by the Affordable Care Act.

b Self-pay or out-of-pocket, when no insurance is billed.

#### Telehealth Encounter Variables

Many included patients had more than 1 telehealth encounter with the health system in the study period; for the scope of this study, we selected each patient’s single most recent telehealth encounter to analyze. For each patient’s most recent telehealth encounter, we extracted telehealth modality, type of provider for the visit, and specialty area or clinic. There were 94 unique specialties represented in encounters; to allow tests of association, the research team condensed these into 5 categories (Table 1). Telehealth modality was either video or telephone.

Telephone visits were charted as “scheduled telephone” or “telephone” encounters; the latter are unscheduled billable calls from providers to patients. Analysis of unscheduled telephone encounters showed that they represented an important telehealth service (see Telehealth Modality by Rurality, Demographic, and Telehealth Encounter Characteristics section), and we therefore elected to retain them in our analysis. Although scheduled and unscheduled telephone encounters may differ in content, we collapsed these categories in order to support tests of association for telephone and video modalities. Unscheduled telephone encounters made up a small proportion of all telehealth encounters, and small cell counts for unscheduled encounters violated the assumptions of chi-square tests.

### Data Analysis

Statistical analysis was conducted from July 1 to October 17, 2023. We conducted descriptive statistical analyses of all patient demographic and telehealth encounter variables, with results presented as distributions for all categorical variables and measures of central tendency for the only continuous variable, patient age. Descriptive statistics were calculated for the total sample by rurality level and by telehealth modality.

Pearson chi-square test or Fisher exact test was used as appropriate to assess for significant associations between categorical variables. Because age was negatively skewed in this sample, the Kruskal-Wallis *H* test was used to test associations with continuous age.

Data were analyzed with Stata BE (version 17.0; StataCorp). For this study, statistical significance was determined at *P* values <.05.

## Results

### Sample Population

There were 9359 unique patients with an address in a rural California zip code who had at least 1 telehealth encounter with the health system from December 2021 to December 2022 (Table 2). Our data were complete for all variables and did not contain missing values. The majority lived in large rural town zip codes (n=6393, 68.3%), 16.5% (n=1543) lived in small rural town zip codes, and 15.2% (n=1423) lived in isolated rural town zip codes. Of 506 rural zip codes in California, 331 (65.4%) were represented in the sample. One quarter of patients (n=2358, 25.2%) lived in just 6 zip codes, which were all large rural towns, and 50.7% (n=4745) of patients lived in 21 zip codes, of which 19 were large rural and 2 small rural zip codes.

**Table 2.** Demographic and most recent telehealth encounter characteristics of all patients residing in rural zip codes^a^ with at least 1 telehealth visit^b^ in the period December 2021 to December 2022, presented by level of rurality^c^.

Characteristic	Total	Large rural town	Small rural town	Isolated rural town	Chi-square (*df*)^d^	*P* value
Level of rurality, n (%)	9359 (100)	6393 (68.3)	1543 (16.5)	1423 (15.2)	N/A^e^	N/A
Sex, n (%)					3.2 (2)	.21
Female	5158 (55.1)	3529 (55.2)	825 (53.5)	804 (56.5)		
Male	4175 (44.6)	2844 (44.5)	717 (46.5)	614 (43.2)
Total^f^	9333 (99.7)	6373 (99.7)	1542 (99.9)	1418 (99.7)
Age (years)					52.2 (2)^g^	<.001^h^
Mean (SD)	56.1 (17.0)	55.2 (17.2)	57.7 (16.5)	58.5 (16.0)		
Median (IQR)	59.4 (42.8-69.4)	58.4 (41.3-69.1)	60.7 (45.7-69.6)	62.1 (47.5-70.5)
Age group (years), n (%)					18.3 (2)	<.001^h^
18‐64	5874 (62.7)	4102 (64.2)	937 (60.7)	835 (58.7)		
65+	3485 (37.2)	2291 (35.8)	606 (39.3)	588 (41.3)
Total	9359 (100)	6393 (100)	1543 (100)	1423 (100)
Race or ethnicity, n (%)					83.9 (6)	<.001^h^
Latinx	1352 (14.5)	1004 (15.7)	251 (16.3)	97 (6.8)
White	6508 (69.5)	4351 (68.1)	1056 (68.4)	1101 (77.4)
Other race or ethnicity	951 (10.2)	664 (10.4)	147 (9.5)	140 (9.8)
Unknown or declined	548 (5.9)	374 (5.8)	89 (5.8)	85 (6)
Total	9359 (100)	6393 (100)	1543 (100)	1423 (100)
Preferred language, n (%)					—^i^	<.001^h^
English	8926 (95.4)	6082 (95.1)	1443 (93.5)	1401 (98.5)		
Spanish	383 (4.1)	279 (4.4)	87 (5.6)	17 (1.2)
Other	50 (0.5)	32 (0.5)	13 (0.8)	5 (0.3)
Total	9359 (100)	6393 (100)	1543 (100)	1423 (100)
Payer, n (%)					40.8 (4)	<.001^h^
Medicare	4193 (44.8)	2749 (43)	742 (48.1)	702 (49.3)		
Other insurance	3304 (35.3)	2293 (35.9)	560 (36.3)	451 (31.7)
Medi-Cal^j^	1862 (19.9)	1351 (21.1)	241 (15.6)	270 (19)
Total	9359 (100)	6393 (100)	1543 (100)	1423 (100)
Patient portal activation, n (%)					2.2 (4)	.69
Activated	8577 (91.6)	5851 (91.5)	1411 (91.5)	1315 (92.4)		
Pending activation	693 (7.4)	481 (7.5)	119 (7.7)	93 (6.5)
Inactivated	89 (1)	61 (1)	13 (0.8)	15 (1.1)
Total	9359 (100)	6393 (100)	1543 (100)	1423 (100)
Provider of most recent telehealth encounter, n (%)					15.7 (6)	.01^h^
Physician	7200 (77)	4192 (76.8)	1176 (76.2)	1112 (78.1)		
Nurse practitioner	1194 (12.8)	829 (13)	202 (12.1)	163 (11.5)
Other providers	500 (5.3)	363 (5.7)	69 (4.5)	68 (4.8)
Physician assistant	465 (5)	289 (4.5)	96 (6.2)	80 (5.6)
Total	9359 (100)	6393 (100)	1543 (100)	1423 (100)
Specialty of most recent telehealth encounter, n (%)					14.7 (8)	.07
Medical specialties	4360 (46.6)	3001 (46.9)	713 (46.2)	646 (45.4)		
Surgical specialties	2692 (28.8)	1822 (28.5)	460 (29.8)	410 (29.8)
Oncology and cancer center	1763 (18.8)	1167 (18.3)	302 (19.6)	294 (20.7)
Women’s, maternal, and fetal specialties	427 (4.6)	317 (5)	52 (3.4)	58 (4.1)
Primary care	117 (1.2)	86 (1.6)	16 (1)	15 (1)
Total	9359 (100)	6393 (100)	1543 (100)	1423 (100)

a Zip code rurality designated using the Washington, Wyoming, Alaska, Montana, Idaho Rural Health Research Center’s zip code approximations of the United States Department of Agriculture’s Economic Research Service Census tract-based Rural-Urban Commuting Area codes.

b Including all visits categorized as video visit, scheduled telephone encounter, telemedicine, and telephone.

c Levels of rurality from least rural (ie, most populous) to most rural: large rural city or town (micropolitan) focused, small rural town focused, and isolated small rural town focused [38]. Patients were grouped by zip code.

d Association with level of rurality was assessed with chi-square test, Fisher exact test, or Kruskal-Wallis *H* test, as appropriate.

e N/A: not applicable.

f The sex categories “unspecified” and “nonbinary” were excluded from the analysis due to small size.

g Chi-square value with ties from the Kruskal-Wallis *H* test of association, for continuous age at first telehealth encounter with level of rurality.

h Significant *P* values.

i Fisher exact, 2-tailed.

j California’s State Medicaid program.

### Demographic and Telehealth Encounter Characteristics by Rurality

Mean age, dichotomous age, race or ethnicity, preferred language, payer, and encounter provider were all significantly associated with patient level of rurality (Table 2). Sex, patient portal activation, and encounter specialty were not significantly associated with rurality. The mean age of the sample was 56.1 (SD 17.0; median 59.4, IQR 42.8-69.4) years and increased as rurality increased. While patients aged 65 years or older made up 37.2% (n=3485) of the sample, the proportion of those older than 65 years was 39.3% (n=606) and 41.3% (n=588) in small rural town and isolated rural town zip codes, respectively.

The majority of rural telehealth patients (n=6508, 69.5%) were White, while 14.5% (n=1352) were Latinx, and 10.2% (n=951) were another race or ethnicity. Race or ethnicity categories represented in the category combined other included Asian (n=142, 1.5%), Black or African American (n=105, 1.1%), Native American or Alaska Native (n=161, 1.7%), and other (n=340, 3.6%). Isolated rural town zip codes had less racial or ethnic diversity: 77.4% (n=1101) of patients in these zip codes were White. English was the preferred language for 95.4% (n=8926) of the sample, with 4.1% (n=383) of telehealth patients preferring Spanish, and 0.5% (n=50) preferring 1 of 21 other languages. Aligning with the Latinx race or ethnicity, notably fewer primary Spanish speakers were in the isolated rural town grouping (n=17, 1.2%).

At 44.8% (n=4193) of the sample, the largest payer group was Medicare, followed by other insurance at 35.3% (n=3304) and Medi-Cal at 19.9% (n=1862) of the sample. Nearly a quarter (n=975, 23.3%) of Medicare recipients were aged 18 to 64 years. There were more Medicare recipients in small rural towns and isolated rural towns (n=742, 48.1% and n=702, 49.3%, respectively), while the isolated rural town grouping had notably fewer other insurance patients (n=451, 31.7% compared to n=3304, 35.3% overall).

### Telehealth Modality by Rurality, Demographic, and Telehealth Encounter Characteristics

Mean age, dichotomous age, race or ethnicity, preferred language, payer, patient portal activation, encounter provider, and encounter specialty were all significantly associated with telehealth modality (Table 3). The level of rurality and sex were not significantly associated with modality.

**Table 3.** Modality (telephone or video) of most recent telehealth encounter by patient demographic and telehealth encounter characteristics for all patients residing in rural zip codes^a^ with at least 1 telehealth visit^b^ in the period December 2021 to December 2022.

Characteristic	Video	Telephone	Total	Chi-square (*df*)^c^	*P* value
Telehealth visit modality, n (%)	8690 (92.9)	669 (7.1)	9359 (100)	N/A^d^	N/A
Level of rurality^e^, n (%)				2.4 (2)	.30
Large rural town	5954 (93.1)	439 (6.9)	6393 (68.3)		
Small rural town	1423 (92.2)	120 (7.8)	1543 (16.5)
Isolated rural town	1313 (92.3)	110 (7.7)	1423 (15.2)
Total	8690 (92.9)	669 (7.1)	9359 (100)
Sex, n (%)				3.3 (1)	.07
Female	4812 (93.3)	346 (6.7)	5158 (55.1)		
Male	3854 (92.3)	321 (7.7)	4175 (44.6)
Total^f^	8666 (92.9)	667 (7.1)	9333 (99.7)
Age (years)				32.8 (1)^g^	<.001^h^
Mean (SD)	55.8 (17.0)	59.6 (16.2)	56.1 (17.0)		
Median (IQR)	59.0 (42.4-69.1)	63.0 (49.0-72.1)	59.4 (42.8-69.4)
Age group (years), n (%)				19.3 (1)	<.001^h^
18‐64	5507 (93.8)	367 (6.3)	5874 (62.8)		
65+	3183 (91.3)	302 (8.7)	3485 (37.2)
Total	8690 (92.9)	669 (7.1)	9359 (100)
Race or ethnicity, n (%)				12.0 (3)	.008^h^
Latinx	1229 (90.9)	123 (9.1)	1352 (14.4)
White	6078 (93.4)	430 (6.6)	6508 (69.5)
Other	881 (92.6)	70 (7.4)	951 (10.2)
Unknown or declined	502 (91.6)	46 (8.4)	548 (5.9)
Total	8690 (92.9)	669 (7.1)	9359 (100)
Preferred language, n (%)				—^i^	<.001^h^
English	8305 (93)	621 (7)	8926 (95.4)		
Spanish	336 (87.7)	47 (12.3)	383 (4.1)
Other	49 (98)	1 (2)	50 (0.5)
Total	8690 (92.9)	669 (7.1)	9359 (100)
Payer, n (%)				27.9 (2)	<.001^h^
Medicare	3843 (91.7)	350 (8.4)	4193 (44.8)		
Other insurance	3130 (94.7)	174 (5.3)	3304 (35.3)
Medi-Cal^j^	1717 (92.2)	145 (7.8)	1862 (19.9)
Total	8690 (92.9)	669 (7.1)	9359 (100)
Patient portal activation, n (%)				219.7 (2)	<.001^h^
Activated	8062 (94)	515 (6)	8577 (91.6)		
Pending activation	547 (78.9)	146 (21.1)	693 (7.4)
Inactivated	81 (91)	8 (9)	89 (1)
Total	8690 (92.9)	669 (7.1)	9359 (100)
Provider of most recent telehealth encounter, n (%)				292.9 (3)	<.001^h^
Physician	6799 (94.4)	401 (5.6)	7200 (76.9)		
Nurse practitioner	1095 (91.7)	99 (8.3)	1194 (12.8)
Other providers	371 (74.2)	129 (25.8)	500 (5.3)
Physician assistant	425 (91.4)	40 (8.6)	465 (5)
Total	8690 (92.9)	669 (7.1)	9359 (100)
Specialty of most recent telehealth encounter, n (%)				—^i^	<.001^h^
Medical specialties	4041 (92.7)	319 (7.3)	4360 (46.6)		
Surgical specialties	2469 (91.7)	223 (8.3)	2692 (28.8)
Oncology and cancer center	1670 (94.7)	93 (5.3)	1763 (18.8)
Women’s, maternal, and fetal specialties	395 (92.5)	32 (7.5)	427 (4.6)
Primary care	115 (98.3)	2 (1.7)	117 (1.2)
Total	8690 (92.9)	669 (100)	9359 (100)

a Zip code rurality designated using the Washington, Wyoming, Alaska, Montana, Idaho Rural Health Research Center’s zip code approximations of the United States Department of Agriculture’s Economic Research Service Census tract-based Rural-Urban Commuting Area codes.

b Including all visits categorized as video visit, scheduled telephone encounter, telemedicine, and telephone.

c Association with modality assessed with chi-square test, Fisher exact test, or Kruskal-Wallis *H* test, as appropriate.

d N/A: not applicable.

e Levels of rurality from least rural (ie, most populous) to most rural: large rural city or town (micropolitan) focused, small rural town focused, and isolated small rural town focused [38]. Patients were grouped by zip code.

f The sex categories “unspecified” and “nonbinary” were excluded from the analysis due to small size.

g Chi-square value with ties from the Kruskal-Wallis *H* test of association, for continuous age at first telehealth encounter with level of rurality.

h Significant *P* values.

i Fisher exact, 2-tailed.

j California’s State Medicaid program.

Most of the telehealth encounters were video visits, at 92.9% (n=8690) of encounters. Before collapsing telephone encounter types, unscheduled telephone encounters comprised 0.7% (69/9359) of included telehealth encounters; 10.3% (69/669) of telephone encounters were unscheduled. The majority (60/69, 87%) of unscheduled telephone encounters were with “resource” providers, a broad category of providers that included counselors and chaplains.

Video users were younger than telephone patients, with a mean age of 55.8 (SD 17.00; median 59.0, IQR 42.4-69.1) years compared to 59.6 (SD 16.2; median 63.0, IQR 49.0-72.1). Patients 65 years or older had 8.7% (n=302) of their telehealth encounters as telephone compared to only 6.3% (n=367) of those younger than 65 years. Telehealth modality also differed substantially by race or ethnicity. Telephone use was highest among Latinx patients (n=123, 9.1%), 2 percentage points higher than the sample and 2.5 percentage points higher than among White patients (n=430, 6.6%). Preferred Spanish language speakers had nearly double the telephone use compared to preferred English language patients, at 12.3% (n=47) and 7% (n=621), respectively.

Medicare patients had the highest use of telephone modality, followed by Medi-Cal patients and patients with other insurance. Patient portal status was strongly associated with telehealth encounter modality (*χ* ^2^_2_=219.7; *P*<.001). Patients with activated portals had only 6% (n=515) of their encounters as telephone, while those with portals that were pending activation had 21.1% (n=146) of their encounters as telephone.

### Patient Portal Activation Status

Patient portal activation status was significantly associated with sex, mean age, dichotomous age, preferred language, and payer (Table 4). We were not able to test the associations of portal activation to race or ethnicity, provider, and specialty due to small cell counts. A large majority of the sample (n=8577, 91.6%) had activated patient portals, while 7.4% (n=693) were pending activation, and 1% (n=89) were inactivated. Patient portal activation was higher for patients who were of female sex, younger than 65 years of age, of White race or ethnicity, primary English speakers, and not on public insurance. These differences were particularly pronounced among Latinx patients (n=1183, 87.5% activated patient portals compared to n=8577, 91.6% overall) and patients who were primary Spanish speakers (n=295, 77% activated patient portals). There was some variation in patient portal activation across provider types, ranging from 89.8% (other providers: n=449) to 93.5% (both nurse practitioners: n=1116 and physician assistants: n=435).

**Table 4.** Electronic patient portal activation status by patient demographic and telehealth encounter characteristics for all patients residing in rural zip codes^a^ with at least 1 telehealth visit^b^ in the period December 2021 to December 2022.

Characteristic	Activated	Pending	Inactivated	Total	Chi-square (*df*)^c^	*P* value
Activation status, n (%)	8577 (91.6)	693 (7.4)	89 (1)	9359 (100)	N/A^d^	N/A
Sex, n (%)					37.8 (2)	<.001^e^
Female	4806 (93.2)	318 (6.2)	34 (0.6)	5158 (55.1)		
Male	3746 (89.7)	374 (9)	55 (1.3)	4175 (44.6)		
Total^f^	8552 (91.6)	692 (7.4)	89 (1)	9333 (99.7)		
Age (years)					35.7 (2)^g^	<.001^e^
Mean (SD)	55.9 (16.9)	56.9 (18.2)	66.3 (11.7)	56.1 (17.0)		
Median (IQR)	59.1 (42.6-69.2)	60.4 (43.4-70.5)	67.2 (60.6-74.0)	59.4 (42.8-69.4)		
Age group (years), n (%)					14.2 (2)	.001^e^
18‐64	5415 (92.2)	419 (7.1)	40 (0.7)	5874 (62.8)		
65+	3162 (90.7)	274 (7.9)	49 (1.4)	3485 (37.2)		
Total	8577 (91.6)	693 (7.4)	89 (1)	9359 (100)		
Race or ethnicity, n (%)					—^h^	N/A
Latinx	1183 (87.5)	158 (11.7)	11 (0.8)	1352 (14.5)		
White	6099 (93.7)	342 (5.3)	67 (1)	6508 (69.5)		
Other race or ethnicity	859 (90.3)	83 (8.7)	9 (1)	951 (10.2)		
Unknown or declined	436 (79.6)	110 (20.1)	2 (0.4)	548 (5.9)		
Total	8577 (91.6)	693 (7.4)	89 (1)	9359 (100)		
Preferred language, n (%)					—^i^	<.001^e^
English	8241 (92.3)	599 (6.7)	86 (1)	8926 (95.4)		
Spanish	295 (77)	86 (22.5)	2 (0.5)	383 (4.1)		
Other	41 (82)	8 (16)	1 (2)	50 (0.5)		
Total	8577 (91.6)	693 (7.4)	89 (1)	9359 (100)		
Payer, n (%)					106.3 (4)	<.001^e^
Medicare	3802 (90.7)	336 (8)	55 (1.3)	4193 (44.8)		
Other insurance	3140 (95)	142 (4.3)	22 (0.7)	3304 (35.3)		
Medi-Cal^j^	1635 (87.8)	215 (11.6)	12 (0.6)	1862 (19.9)		
Total	8577 (91.6)	693 (7.4)	89 (1)	9359 (100)		
Provider of most recent telehealth encounter, n (%)					—^h^	N/A
Physician	6577 (91.3)	553 (7.7)	70 (1)	7200 (76.9)		
Nurse practitioner	1116 (93.5)	66 (5.5)	12 (1)	1194 (12.8)		
Other providers	449 (89.8)	48 (9.6)	3 (0.6)	500 (5.3)		
Physician assistant	435 (93.5)	26 (5.6)	4 (0.9)	465 (5)		
Total	8577 (91.6)	693 (7.4)	89 (1)	9359 (100)		
Specialty of most recent telehealth encounter, n (%)					—^h^	N/A
Medical specialties	3977 (91.7)	324 (7.4)	39 (0.9)	4360 (46.6)		
Surgical specialties	2421 (89.9)	253 (9.4)	18 (0.7)	2692 (28.8)		
Oncology and cancer center	1640 (93)	91 (5.2)	32 (1.8)	1763 (18.8)		
Women’s, maternal, and fetal specialties	402 (94.2)	25 (5.6)	0 (0)	427 (4.6)		
Primary care	117 (100)	0 (0)	0 (0)	117 (1.2)		
Total	8577 (91.6)	693 (7.4)	89 (1)	9359 (100)		

a Zip code rurality designated using the Washington, Wyoming, Alaska, Montana, Idaho Rural Health Research Center’s zip code approximations of the United States Department of Agriculture’s Economic Research Service Census tract-based Rural-Urban Commuting Area codes.

b Including all visits categorized as video visit, scheduled telephone encounter, telemedicine, and telephone.

c Association with modality assessed with chi-square test, Fisher Exact test, or Kruskal-Wallis *H* test, as appropriate.

d N/A: not applicable.

e Significant *P* values.

f The sex categories “unspecified” and “nonbinary” were excluded from the analysis due to small size.

g Chi-square value with ties from the Kruskal-Wallis *H* test of association, for continuous age at first telehealth encounter with level of rurality

h Not available: chi-square analysis not appropriate due to small cell counts, and our statistical software could not execute Fisher exact test with this number of variable categories.

i Fisher exact, 2-tailed.

j California’s State Medicaid program.

## Discussion

### Principal Findings

In this study, we used 3 levels of rurality to characterize a population of rural-dwelling California adults who used telehealth services at an urban medical center from December 2021 to December 2022. Patients who lived in more rural zip codes were older, and a much higher proportion were White and primary English speakers. This aligns with other research showing that rural populations tend to be on average older and less racially and ethnically diverse [3,4]. Older age among more rural patients is of particular concern, as challenges associated with more rural status (eg, distance to services and weather disruptions) may be more impactful for older adults, compounding health care access challenges. Older adults also have lower digital access [41] and higher telehealth unreadiness [42], evidenced in our study by fewer video visits and lower patient portal use among older patients. Older patients may, in part, prefer telephone visits over video due to lower digital comfortability. Interventions to increase health care access through telehealth use among rural older adults could include digital access and comfortability assessments, digital education, and support for rural connectivity.

A quarter of our sample was comprised of patients from race or ethnicity groups other than White, in line with the rural United States as a whole [43]. However, at the time of the 2020 US Census [44], rural California counties [45] had a higher percentage of residents who were American Indian or Alaska Native (5.6%), Asian (2.1%), Black or African American (1.7%), and Hispanic or Latino (22.8%) than in our sample. While these data do not support a direct comparison because of different rurality measures (counties are the smallest scale for which US Census data are consistently available, as the US Census Bureau QuickFacts data tool provides statistics only for counties and for cities and towns with a population of 5000 or more), this may indicate that fewer rural individuals from these race or ethnicity groups are using telehealth at this urban health center. This is significant, given evidence that rural American Indian or Alaska Native and populations of color experience worse health outcomes than rural White populations [6,7,46]. Rural American Indian or Alaska Native and populations of color contend with complex barriers to realizing health as a result of legacies of colonization and slavery [6,47]. For these populations, patient-centeredness and cultural tailoring [48] will be of central importance for successful implementation and equitable use of telehealth services.

Our findings align with existing research showing higher video visit use by White patients compared to patients of other races or ethnicities [29-32]. In our rural sample, patients who were Latinx had the lowest video visit use despite being younger and living less rurally. These findings agree with a majority of studies showing lower video use among Hispanic or Latino patients [30,31,39], although Drake et al [29] found higher video use among rural and urban Hispanic patients in North Carolina. Research has also found that while Hispanic or Latino individuals used less video visits, they had higher overall telehealth use compared to non-Hispanic White individuals [24,32,49]. We did not include a comparison to in-person patients at the health center, and more research is needed to explore how rural Latino patients use in-person versus telehealth specialty services at distant health centers.

Multiple other studies have shown that patients with limited English proficiency (LEP) have fewer video and more telephone visits than English-proficient patients [28,30,31,33]. While these studies contained mixed rural-urban samples, we confirmed this finding in our entirely rural sample, where video use disparity was greatest among Spanish-speaking patients. Patients with LEP experience multiple barriers to health care access overall and, consequently, worse health outcomes [50]. These barriers may be confounded in rural areas with fewer services and lower diversity. Video visit disparities may exacerbate this issue. While video access is limited by patient-level LEP barriers, such as mistrust and perceived discrimination [51,52], clear provider- and system-level barriers also exist. Patients with LEP may not be offered video visits [25,33], a lack of language-concordant front office staff poses challenges to patients with LEP in obtaining appointments [51] and coordinating care [52], and difficulties bringing an interpreter on video platforms may also deter providers from offering video visits to patients with LEP [25,53]. Integrated video translation services, LEP community outreach, and digital access assessment, as well as the availability of language-concordant outreach materials, front office or call center staff, and patient portals have all been identified as important areas for intervention [50,53].

The patterns we found of lower video visit use among older patients, Latinx patients, and Spanish-speaking patients are similar to those reported in studies early in the COVID-19 pandemic [28-31]. Our video use findings also concur with more recent national data on older patients and Latinx patients [32]. The persistence of video visit disparities after the initial phases of the COVID-19 PHE, when systems- and patient-level telehealth barriers were likely highest, related to implementation and scale-up challenges, underscores the need for ongoing research and policy attention to understand this issue. As others have noted [25,27,28], telephone visits likely support overall access for vulnerable populations; therefore, while efforts should be made to address video barriers, policy should continue to support telephone visit availability and reimbursement.

As a proxy measure of digital engagement, an inactive patient portal may indicate patients at risk of digital access disparities [54,55], and our findings appear to substantiate this. Video visits were less common among patients whose portals were inactive or pending activation than among those with active portals, a finding we anticipated based on other studies [31,34,39]. On the other hand, our finding that neither telehealth modality nor patient portal status was significantly associated with rurality level was novel. Previous research has found that rural patients were significantly less likely to have video visits [28,34] and significantly less likely to have an activated patient portal [39]. However, another study found that while rurality was not associated with 3 measures of technology access, video and portal use were both positively associated with living in isolated rural census tracts [40].

In this context, our findings contribute to a complex picture of digital access and telehealth use patterns among rural populations. Our paper contributes to understanding telehealth modality use between patients living at different levels of rurality. One potential explanation for our finding of no association is that these other studies used nonrural comparison groups, while our sample was entirely rural. Another possible explanation is the use of different methodologies to define rurality, for example, RUCA codes versus Rural-Urban Continuum Codes, as well as different geographic units, such as census tract, zip code, or county [56,57]. Finally, rural populations in the United States are heterogeneous [8,43,58], and these findings may represent meaningful variation between these rural populations.

### Implications for Research and Policy

Our findings point to several areas for future research and intervention. More study is needed to fully characterize rural telehealth users, specifically rural populations of color. Future studies should apply sampling methods that account for the relatively fewer people of color living in rural areas to support statistical analysis of these groups. Research is also needed to elucidate the nature of relationships between patient factors and telehealth modality use among rural patients using telehealth at distant urban medical centers. For example, research should explore how patient preference for telehealth modality relates to age, race or ethnicity, preferred language, and educational attainment, with attention to how factors such as perceived lack of access or discrimination impact modality preferences among these groups.

Policy interventions are needed to support equitable access to telehealth overall [59] as well as the appropriate application of telephone and video visit modalities [27,28]. To support access for marginalized patient populations, such interventions should include the development of culturally tailored and language-concordant telehealth patient outreach and education. Centering patient perspectives in the development of these resources, through research participation and patient advisory boards, can help ensure that the materials are accurately targeted to address the perceived needs and preferences of their intended recipients [48,59]. Patients from marginalized groups may also prefer telephone visits over video due to concerns of discrimination or lack of access [47,51,52]; culturally tailored outreach and patient advisory boards may particularly help address these concerns. For older adults, as noted, a preference for the telephone over video may arise from uncertainty around digital devices. Policy to address equitable use of video visits should therefore support assessments of not just patient digital access but also digital comfort and related patient digital education. Providers can advocate for policies such as these to be implemented within their clinics or health systems. However, to ensure appropriate video access, policies from state and federal governments must also address structural access barriers. Policy support is needed for the ongoing development of broadband access in rural and other underserved areas [60,61], free or low-cost smart device and data plans for low-income patients, and the development of telehealth resources in public spaces, such as rural libraries [62]. Providers can support structural policy changes by advocating with professional organizations or with state and federal representatives.

Finally, policymakers must stay abreast of evidence regarding the effectiveness and accessibility of telephone and video telehealth modalities to inform reimbursement decisions in support of equitable health care access. Reimbursement policy can address important provider-side telehealth barriers, for example, by incentivizing video visits or by providing payment parity for telehealth and in-person services, thereby encouraging providers to direct resources to telehealth infrastructure, workflows, and training. The recent decision to end most CMS Medicare telehealth waivers deployed under COVID-19 by October 2025, if not reversed, will likely undermine health care access overall for older patients, particularly lower-income and rural populations. The exception for rural areas is not yet well-defined. Requiring rural Medicare patients to return to clinics to access telehealth services will entail a substantial travel burden for many older rural residents [63], and the loss of audio-only reimbursement may eliminate one of the most accessible access options for patients. Further, given the role Medicare reimbursement plays in informing reimbursement decisions across payer types, the loss of CMS waivers may signal the loss of telehealth coverage for privately insured individuals as well.

### Limitations

Low representation of patients from several race or ethnicity groups in our sample and the choice to collapse several categories of race or ethnicity to enable tests of association were limitations of our study. The categories of race or ethnicity we combined represent distinct populations of rural residents, who experience particular structural barriers to realizing health, and focused research with these patient populations is needed. Compared to the population of rural California counties overall [44,45], our sample was comprised of substantially more female patients (n=5158, 55.1% vs 48.5% overall) and more patients aged 65 years or older (n=3485, 37.2% vs 25.8% overall). This may limit the generalizability of our findings to diverse rural patient populations, rural male patients, and rural patients younger than 65 years of age. In our data, services provided by nurse practitioners, physician assistants, or other provider types may have been billed under the physician billing code, potentially inflating the number of physician encounters. Further, while patient portal activation status and race or ethnicity showed a strong trend of association, we were not able to test this due to limitations in our statistical analysis. As discussed, this proxy measure could not fully capture broadband access or digital literacy data, and related findings should be generalized with caution. Future research could use Federal Communications Commission county-level broadband availability data and measures of patient personal device use to more accurately capture digital access. Our data also did not support comparison to rural in-person patients. Self-selection bias may have been present, as there may be systematic differences between patients who opted for telehealth and those who chose in-person care. Data were not available on the health system’s total rural population. Further research is needed to explore how these groups differ in the use of specialty care at an urban medical center. Finally, our data are from a single health system; while we recognize the limitations of a dataset from a single health system, we believe findings may be applicable to similar large urban health systems nationally, insofar as they often provide specialty care to patients over a broader geographic region.

### Conclusions

In this sample of rural patients who used telehealth at an urban medical center, video visit use and patient portal activation were lower among patients who were older, Latino race or ethnicity, primary Spanish speakers, and publicly insured. Targeted policies are needed to support appropriate video visit use in populations at risk of access barriers, including patient digital access assessment and education, culturally tailored and language-concordant telehealth outreach and education, low-cost smart device and data plans for low-income patients, rural broadband development, and evidence-based telehealth reimbursement policy.

## Abbreviations

CMS: Centers for Medicare and Medicaid Services
EHR: electronic health record
HIPAA: Health Insurance Portability and Accountability Act
LEP: limited English proficiency
PHE: Public Health Emergency
RUCA: Rural-Urban Commuting Area
